# Morphology Controllable Synthesis of NiO/NiFe_2_O_4_ Hetero-Structures for Ultrafast Lithium-Ion Battery

**DOI:** 10.3389/fchem.2018.00654

**Published:** 2019-01-10

**Authors:** Ying Wang, Shengxiang Wu, Chao Wang, Yijing Wang, Xiaopeng Han

**Affiliations:** ^1^School of Chemistry & Materials Science, Jiangsu Key Laboratory of Green Synthetic Chemistry for Functional Materials, Jiangsu Normal University, Xuzhou, China; ^2^Key Laboratory of Advanced Energy Materials Chemistry (MOE), College of Chemistry, Nankai University, Tianjin, China; ^3^School of Materials Science and Engineering, Tianjin Key Laboratory of Composite and Functional Materials, Tianjin University, Tianjin, China

**Keywords:** lithium ion battery, NiO/NiFe_2_O_4_, morphology control, hetero-structure, electrochemical performance

## Abstract

Rational design of high performance anode material with outstanding rate capability and cycling stability is of great importance for lithium ion batteries (LIBs). Herein, a series of NiO/NiFe_2_O_4_ hetero-structures with adjustable porosity, particle size, and shell/internal structure have been synthesized via a controllable annealing process. The optimized NiO/NiFe_2_O_4_ (S-NFO) is hierarchical hollow nanocube that is composed of ~5 nm subunits and high porosity. When being applied as anode for LIBs, the S-NFO exhibits high rate capability and excellent cycle stability, which remains high capacity of 1,052 mAh g^−1^ after 300 cycles at 5.0 A g^−1^ and even 344 mAh g^−1^ after 2,000 cycles at 20 A g^−1^. Such impressive electrochemical performance of S-NFO is mainly due to three reasons. One is high porosity of its hierarchical hollow shell, which not only promotes the penetration of electrolyte, but also accommodates the volume change during cycling. Another is the small particle size of its subunits, which can effectively shorten the electron/ion diffusion distance and provide more active sites for Li^+^ storage. Besides, the hetero-interfaces between NiO and NiFe_2_O_4_ also contribute toitsfast charge transport.

## Introduction

Lithium ion batteries (LIBs), with the advantages of high energy density and environmental benignity, have become the most widely used energy storage systems for portable electronic equipment. The growing needs for high-performance electronic devices and electric vehicles, however, constantly demand LIBs for further innovation, in terms of higher energy/power density, longer lifetime, greater rate capability, and lower cost. It is therefore crucial to develop high-performance electrode materials (Poizot et al., 2000; Goriparti et al., 2014; Peng et al., 2015; Wang et al., 2015a,b; Hou et al., 2016; Pagot et al., 2017; Wei et al., 2018; Xu et al., 2018; Zhang et al., 2018; Huang et al., in press; Liu et al., in press), or to develop other energy storage devices, such as sodium ion battery, metal-O_2_ battery (Han et al., 2014, 2017, 2018), and Li-S battery (Kim et al., 2016; Shen et al., 2018). As for the anode materials in LIBs, NiFe_2_O_4_ has drawn extensive attention, due to its low price, earth abundant, and high theoretical capacity (915 mAh g^−1^) (Park et al., 2015; Jin et al., 2017; Lina et al., 2017). During the Li^+^ insertion/extraction, however, NiFe_2_O_4_ suffers from unsatisfied capacity retention and poor rate capability that causes by the server volume change and limited electrochemical kinetics. Although numerous strategies have been proposed to improve the electrochemical performance of NiFe_2_O_4_ (Gao et al., 2017; Jiang et al., 2017; Li D. et al., 2017; Zhou et al., 2017), it is still a big challenge to deliver decent capacities at high current density (above 10 A g^−1^) or well-capacity retention over 2000 cycles.

Smart design of hetero-structures is an emerging strategy to improving the electrochemical kinetics of transition metal oxides (TMOs) (Zheng X. et al., 2018). For example, Hou et al. reported the appealing electrochemical performance of ZnFe_2_O_4_/ZnO, which outperformed the single ZnFe_2_O_4_ (Hou L. et al., 2015). Ting et al. observed the fast charge transfer in the CoO/Cu_2_O hetero-structure, where Li^+^ diffusion kinetics and electronic conductivity were promoted (Tingting et al., 2017). Considering the high theoretical capacity (718 mAh g^−1^) and non-pollution of NiO (Shi et al., 2018; Yin et al., 2018), hybridizing NiO with NiFe_2_O_4_ host great promising for high performance LIBs. However, only limited studies have been paid to the Li storage performance of NiO/NiFe_2_O_4_ hybrid (Du et al., 2016).

Tailoring the nano-architecture of electrode is another important approach to enhance the electrochemical performance. Previous literatures had evidenced that morphology of electrode materials can effectively affect its electrochemical performance (Li et al., 2013; Hou H. et al., 2015; Liang et al., 2018). Particularly, hierarchical hollow electrode have received great attention (Chen et al., 2015; Zheng Z. et al., 2018). Nowadays, TMOs in different morphologies, such as nanotube (Gang et al., 2014; Huang et al., 2014), porous plates (Hui et al., 2016; Wang et al., 2016), and hollow octahedron, have been realized. However, the relationship between morphology of electrode and its electrochemical performance is still under investigation.

Taking all the discussion above into consideration, fabricating NiO/NiFe_2_O_4_ hetero-structure, and further tailoring its morphology seem to have great potential for ultrafast LIBs. Ni_3_[Fe(CN)_6_]_2_, a typical Prussian blue analog, was selected as precursor to synthesize morphology controllable NiO/NiFe_2_O_4_ hetero-structures, since it contains both Fe and Ni element at the same. By taking advantage of the unique reactivity and thermal stability of Ni_3_[Fe(CN)_6_]_2_, herein, we obtain a series of NiO/NiFe_2_O_4_ with adjustable porosity, particle size, and shell/internal structure *via* a simple calcination procedure. The obtained samples are porous filled-nanocubes (P-NFO), hierarchical hollow-nanocubes with ~5 nm subunits (S-NFO), and hierarchical hollow-nanocubes with ~12 nm subunits (L-NFO). The optimized S-NFO exhibits excellent Li^+^ storage performance, with high capacity of ~1,052 mAh g^−1^ after 300 cycles at 5.0 A g^−1^ and even ~344 mAh g^−1^ after 2,000 cycles at 20 A g^−1^. The superior electrochemical performance of S-NFO is attributed to its unique structural advantages, which combined the hetero-structure, high porosity, and small particle size. Further investigation reveals that the electrochemical reaction of S-NFO is dominated by capacitive behavior. The simple and large-scalable synthesis of these NiO/NiFe_2_O_4_ hetero-structures may shed light on design for other high performance electrode materials.

## Experimental

### Synthesis of NiO/NiFe_2_O_4_ Hetero-Structures

NiO/NiFe_2_O_4_ hetero-structures were obtained as follows. First, Ni_3_[Fe(CN)_6_]_2_ (NiFe-PBA) was synthesized according to previous literatures (Xuan et al., 2018) with minor modifications. Second, the NiFe-PBA was transferred into a tube furnace, heated to the target temperature (350, 450, or 550°C) at a heating ramp of 0.5°C min^−1^, and held for 6 h in air atmosphere. The resultant samples were marked as P-NFO, S-NFO, or L-NFO according to their respective morphology.

### Characterization

X-ray diffraction (XRD, Bruker, D8-Advance), field-emission scanning electron microscopy (FESEM, JEOL, SU8010), field-emission transmission electron microscope (FETEM), high-resolution TEM (HR-TEM, Tecnai G2 F20 S-TWIN), and energy dispersive X-ray (EDX) analysis (taken with X-ray spectroscopy attached to the Tecnai G2 F20 S-TWIN), were employed to characterize the phase and morphological structures. Thermogravimetric analysis (TGA, TA-Q50) was performed to analyze the thermal stability. N_2_ adsorption/desorption isotherms (Quantachrome, Autosorb-IQ2-VP) was conducted to obtain the physical surface area and pore distribution. X-ray photoelectron spectroscopy (XPS, Thermo ESCALAB 250XI) were employed to detect the elemental composition and surface oxidation states of synthesized samples.

### Electrochemical Measurements

Electrochemical performance of as-prepared samples were carried out by assembling standard 2,032 coin cells in an argon-filled glove box with the oxygen and water content below 0.1 ppm. Active materials, super conductive carbon black, and poly(vinyldifluoride) (PVDF, Sigma Aldrich) were mixed in a weight ratio of 7:2:1, dispersed in N-methyl-2-Pyrrolidinone (NMP), then milled for 30 min to form a slurry. The slurry was cast onto copper foil using a doctor blade and vacuum dried at 120°C overnight. 1 M LiPF_6_ (Sigma Aldrich) in ethylene carbonate (EC, Sigma Aldrich), diethyl carbonate (DEC, Alfa Aesar), and fluorinated ethylene carbonate (FEC, Sigma Aldrich) (volume ratio 6:3:1) was used as the electrolyte. Polypropylene (PP, MTI Cooperation) was used as the separator. For half-cells, a lithium disc (MTI Corporation) was used as the counter electrode. Galvanostatic charge-discharge tests were carried out at room temperature on a battery testing system (LAND Wuhan, China) in a potential range of 0.01–3.00 V (vs. Li/Li^+^). Cyclic voltammetry (CV) tests and electrochemical impedance spectroscopy (EIS) measurements were performed on a CHI-660E electrochemical work station. For the full-cells, the cathodes were assembled by mixing LiCoO_2_ (Sigma Aldrich) with carbon black, and PVDF in a weight ratio of 8:1:1. The electrolytes and separator in full-cells were same as those in the half-cells. Electrochemical performance of full-cells were tested in a voltage window between 1.0 and 3.9 V. The weight ratio of positive materials to negative materials was designed as 4:1. Specific capacity of all the cells was calculated based on only the mass of active materials in anode.

## Results and Discussion

Crystallographic structure and purity of the NiFe-PBA precursors were studied by XRD (Figure S1a). All the diffraction peaks match well with fcc Ni_3_[Fe(CN)_6_]_2_ (JCPDS No. 86-0501), suggesting the high purity of NiFe-PBA. The SEM image (Figure S1b) shows that NiFe-PBA are uniform nano-cubes with an average size of ~180 nm and smooth surfaces. The synthesis process of NiFe-PBA was referred to previous literatures (Xuan et al., 2018): the formation of these NiFe-PBA cube is caused by coordinate process between Ni^2+^ and [Fe(CN)_6_]^3−^, and dominated by a kinetically controlled process to obtain uniform cubes (Hu et al., 2013). TGA curve (Figure S2) reveals thermal stability of the NiFe-PBA, which starts to decompose at 252 °C in air. Therefore, the NiFe-PBA was heated in air at 350, 450, or 550 °C to obtain the morphology control of NiO/NiFe_2_O_4_ hetero-structures.

**Figure 1:**
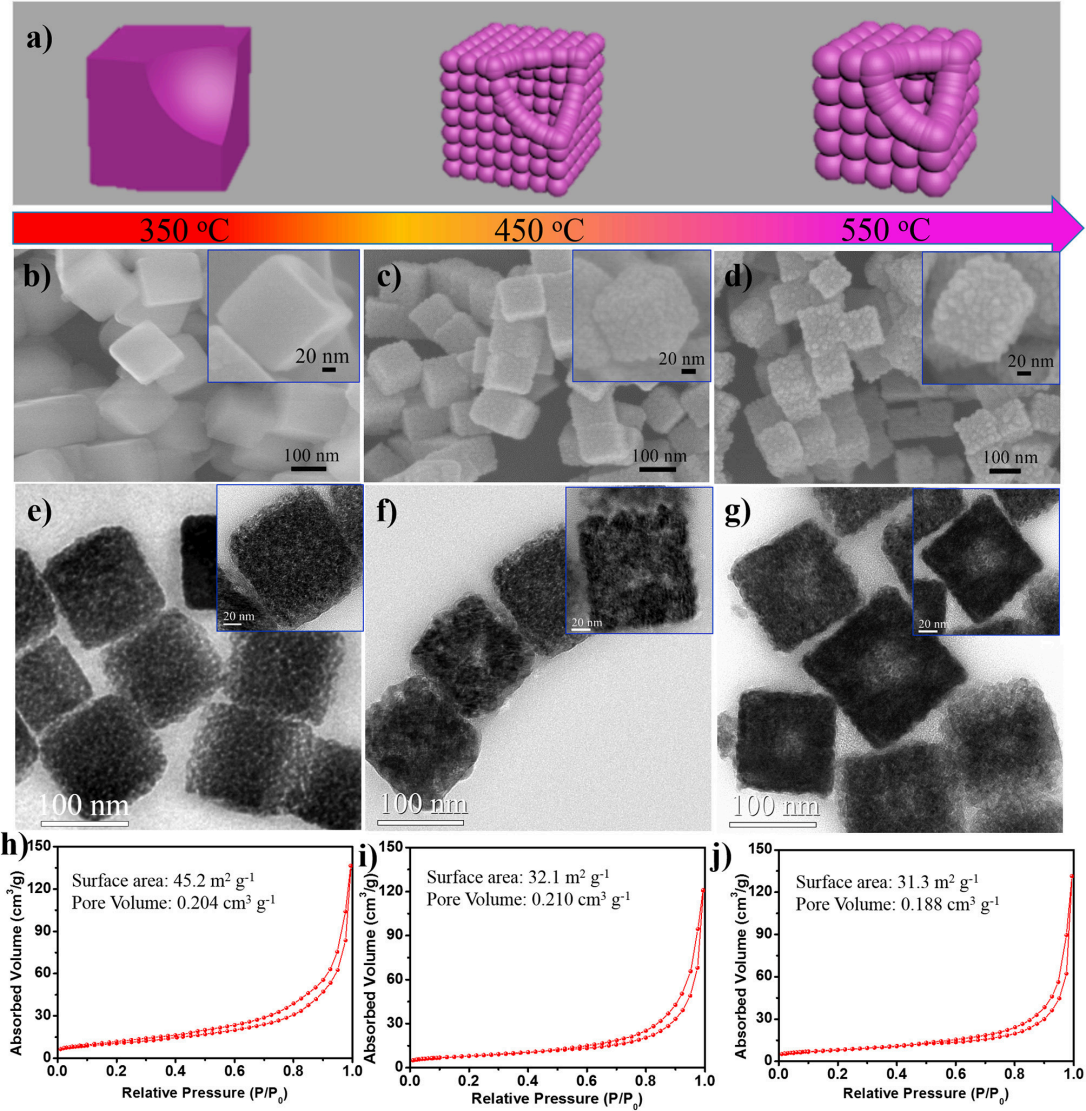
**(a)** Schematic illustration for morphology evaluation of NiO/NiFe_2_O_4_ hetero-structures. **(b–d)** SEM images, **(e–g)** TEM pictures, and **(h–j)** N_2_ isothermals of the P-NFO, S-NFO, and L-NFO, respectively. Insets in **(b–d)** and **(e–g)** are the enlarged SEM and TEM images of an individual P-NFO, S-NFO, and L-NFO, respectively.

The NiO/NiFe_2_O_4_ hetero-structures that collected after controllable annealing at different temperatures show distinct nano-architectures (Figure 1a). The sample that annealed at 350°C well retains the cubic shape of the Ni-Fe PBA with a diameter size of ~130 nm. The magnified SEM image (inset Figure 1b) reveals the integrate surface of the P-NFO sample. When elevated the calcination temperature to 450 °C, the size of cubes shrinks further to ~100 nm and hierarchical surfaces are formed, which consist of about 5 nm nanoparticles (inset of Figure 1c). As for the L-NFO, although hierarchical surfaces are still observed, the subunits grow to a larger size of around 12 nm (inset Figure 1d). TEM images (Figures 1e–g) display more morphological details about the samples. The P-NFO shows a highly porous structure with filled internal. Internal cavities are observed in the S-NFO and L-NFO, confirming the formation of hierarchical hollow-nanocubes. Interestingly, besides the particle size of subunit, the internal cavity also grows larger in the L-NFO compared to that of S-NFO sample. The formation of these distinct NiO/NiFe_2_O_4_ samples is dominated by two major factors: thermally induced oxidation process of the NiFe-PBA, and further growth of the formed NiO/NiFe_2_O_4_ composite (Zhang et al., 2013). In the former, metallic elements in NiFe-PBA react with oxygen to form homogenous NiO/NiFe_2_O_4_ hetero-structure. As the annealing temperature raises to higher value, the rapid mass-transport from core to shell causes the formation of hollow interior in resultant samples (S-NFO and L-NFO) (Guo et al., 2015). Meanwhile, the latter results a further growth of NiO/NiFe_2_O_4_ subunits, that following an Oswald ripening process. Therefore, compared with S-NFO, the L-NFO shows a larger interior hollow and bigger subunit size.

N_2_ adsorption-desorption isothermals (Figures 1h–j) of these samples show a similar type form, exhibiting typical IV with H1 hysteresis loops, indicative of their mesoporous structure. The specific surface area is 45.2, 32.1, and 31.3 m^2^ g^−1^ for P-NFO, S-NFO, and L-NFO sample, respectively. When increasing the annealing temperature, both the particle size of subunits and interior cavity grow to larger values, therefore, the smallest surface area is observed in the L-NFO sample, which is in agreement with the observation in Figure 1. The narrow and small hysteresis loops also suggest co-existence of macropores in all samples. Notably, the combination of mesopores and macropores can facilitate better penetration of electrolyte, which is an important factor for enhancing the electrochemical performance (Jiang et al., 2018). The pore volume of P-NFO, S-NFO, and L-NFO are 0.204, 0.210, and 0.188 cm^3^ g^−1^, respectively. High pore volume of the P-NFO and S-NFO indicates that the good porous feature of NiFe-PBA is well-reserved in both samples. Therefore, NiO/NiFe_2_O_4_ hetero-structures with different porosity, particle size, and shell/internal structures are successfully synthesized *via* a simple controllable annealing process.

**Figure 2:**
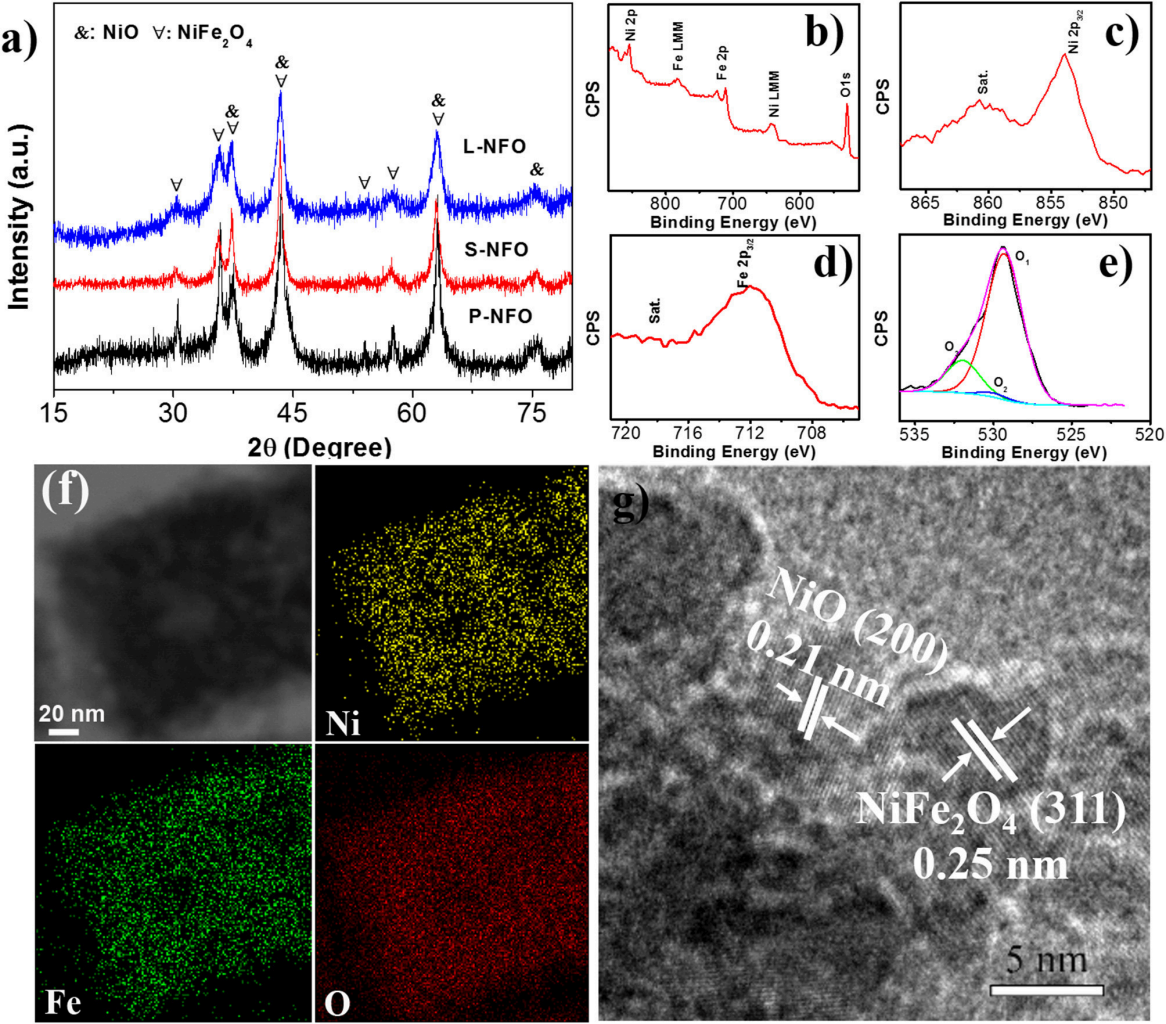
**(a)** XRD patterns of P-NFO, S-NFO, and L-NFO. Chemical and structural investigation of S-NFO: **(b)** XPS spectrum, **(c–e)** high-resolution XPS spectrum for Ni 2p, Fe 2p, and O 1s, **(f)** EDX elemental mapping, and **(g)** HR-TEM images.

Figure 2 presents more structural information about the resultant samples. XRD patterns of the P-NFO, S-NFO, and L-NFO (Figure 2a) match well with crystallographic structure of cubic NiO (JCPDS No. 01-1239) and NiFe_2_O_4_ (JCPDS No. 01-074-2081), confirming their hybrid composition. The XRD pattern of S-NFO was selected for GSAS Rietveld refinement to determine the phase content of NiFe_2_O_4_ and NiO, as shown in Figure S3. According to the refinement result, the phase content of NiFe_2_O_4_ and NiO is determined to be 63 and 37%. The broad diffraction peak of the XRD pattern indicated a smaller particle size. Specifically, the particle size of the P-NFO, S-NFO, and L-NFO is calculated to be 7.67, 5.73, and 13.14 nm, respectively, according to the Scherrer equation based on the peak at 43.4 degree. XPS spectra of the S-NFO are shown in Figures 2b–e. XPS survey reveals the existence of only Ni, Fe, and O elements, suggesting high purity of the S-NFO sample. In the Ni 2p spectrum, the main peak at 855.7 eV (Ni 2p_3/2_) and the satellite peak at 860.7 eV are the typical Ni^2+^ bond in NiO and NiFe_2_O_4_ (Song et al., 2018). In Figure 2d, the dominate peaks at 710.0 eV, together with the satellite peak are the typical Fe 2p_3/2_ signal of NiFe_2_O_4_ (Gao et al., 2017). For the O1s spectrum, peaks locate at 528.9 and 530.0 eV are assigned to the typical metal-oxygen bonds (Jin et al., 2017). The O1s peak at 532.1 eV is most likely associated with defects and under-coordinated lattice oxygen (Qiu et al., 2015; Yuan et al., 2015). Elemental mapping of an isolated S-NFO nanocube is used to investigate its chemical composition (Figure 2f). The hollow structure of S-NFO is further confirmed by the color contrast between shell (darker) and hollow interior (lighter). Clearly, the S-NFO consists of Ni, Fe, and O elements, and all the elements are homogeneous distributed, indicating homo-distribution of NiO/NiFe_2_O_4_ hetero-structures. Taking the Fe mapping for example, the homogenous distribution of Fe in S-NFO is confirmed by the full cover of green dots through the selected cube. Comparing with the intensive part at the edge, these green dots are less intensive in the interior, proving its hollow structural feature. The uniform elemental distribution was also observed in the P-NFO and L-NFO (Figures S4a–f). HR-TEM image in Figure 2g reveals that the S-NFO exhibits a hierarchical structure that is composed of small nanoparticles (~5 nm) as its subunits. The lattice fringes of 0.21 and 0.25 nm can be well indexed to the (200) and (311) lattice planes of cubic NiO and NiFe_2_O_4_, revealing the intimate junction between NiO and NiFe_2_O_4_. The weight ratio of Ni to Fe in S-NFO was determined to 32: 31 by EDS spectrum (Figure S5). Therefore, the S-NFO consists of 65 wt.% NiFe_2_O_4_ and 35 wt.% NiO, similar with the XRD refinement result.

**Figure 3:**
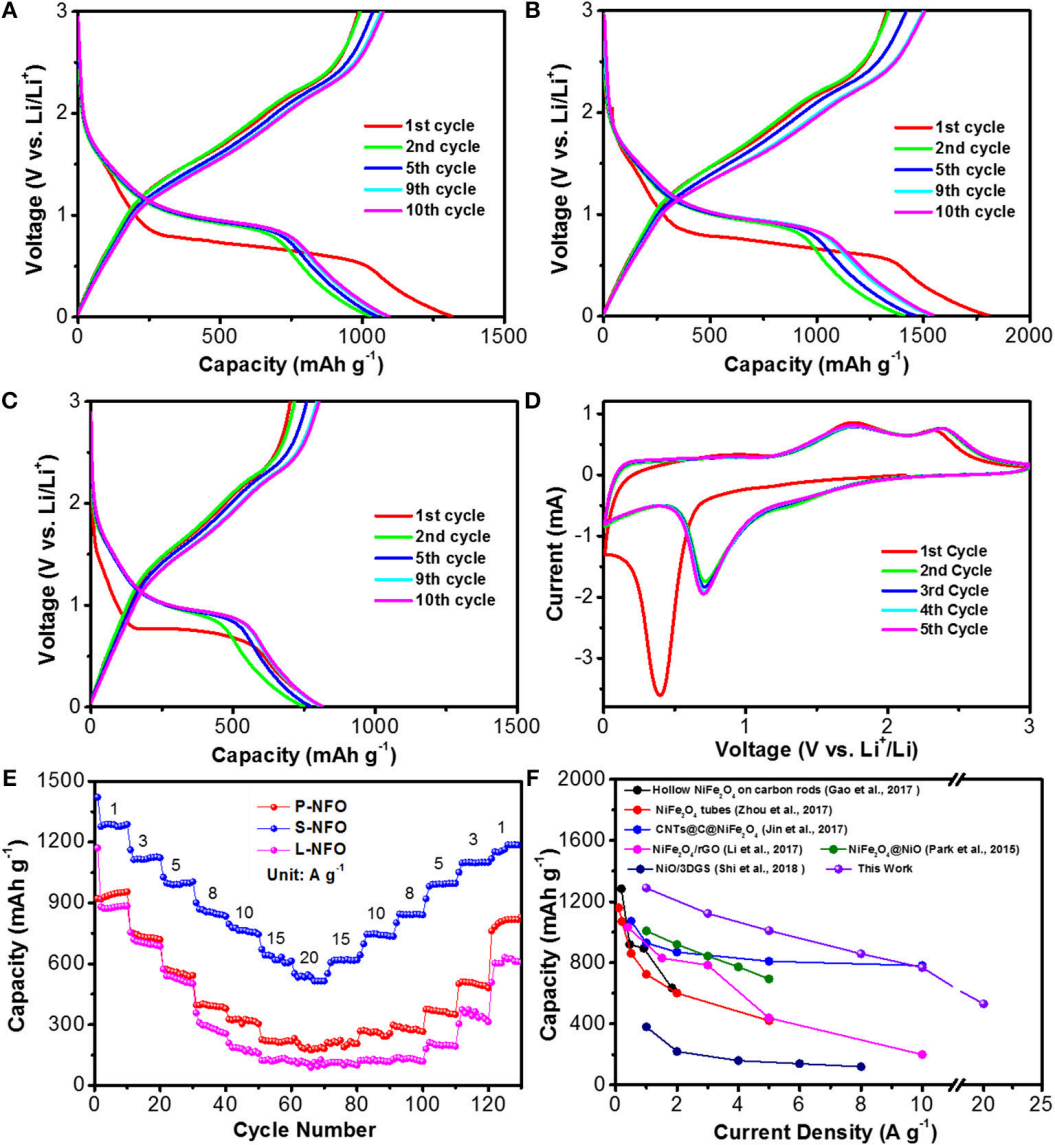
**(A–C)** Charge-discharge profiles of P-NFO, S-NFO, and L-NFO at 100 mA g^−1^, respectively. **(D)** Typical cyclic voltammetry curves of S-NFO at a scan rate of 0.5 mV s^−1^. **(E)** Rate capability of P-NFO, S-NFO, and L-NFO at various current densities from 1.0 to 20 A g^−1^ and then back to 1 A g^−1^. **(F)** Electrochemical performance comparison of the S-NFO with other Ni-based, Fe-based, and hetero-structure materials.

Electrochemical measurements of the NiO/NiFe_2_O_4_ hetero-structures were carried out to clarify the relationship between porosity, particle size and electrochemical performance. The charge-discharge curves of P-NFO, S-NFO, and L-NFO at the first, second, fifth, ninth, and tenth cycle are given in Figures 3A–C. In the first discharge process, all samples exhibit similar profiles with an obvious plateau at ~0.75 V, which shifts to ~1.0 V and remains stable in the subsequent cycles. The upward shifted voltage platform is caused by structural reorganization, new phase formation, and a polarization change of electrodes materials (Zou et al., 2014). The initial discharge capacity of P-NFO, S-NFO, and L-NFO is 1314, 1805, and 813 mAh g^−1^, respectively. The charge profiles are relatively smooth with two bumps at ~1.3 and 2.3 V, corresponding to the oxidation of Ni^0^ and Fe^0^ (Park et al., 2015; Du et al., 2016). The initial charge capacity is 986, 1330, and 701 mAh g^−1^ for the P-NFO, S-NFO, and L-NFO, corresponding to an initial coulombic efficiency (ICE) of 75, 74, and 86%, respectively. The irreversible capacity loss in the first cycle may be attributed to formation of solid electrolyte interface (SEI) film, and irreversible side reactions of the NiO/NiFe_2_O_4_ composites (Park et al., 2015). The higher ICE value of the L-NFO is due to its smaller surface area, which reduces the formation of the SEI film. In the 10th cycle, the P-NFO, S-NFO, and L-NFO show a stable discharge/charge capacity of 1092/1077, 1539/1501, 814/802 mAh g^−1^, respectively. The high capacity of S-NFO [theoretical capacity is 846 mAh g^−1^, 718 × 35% (NiO)+915×65% (NiFe_2_O_4_)] is caused by the extra contribution of its nano-subunits, and other factors such as kinetic limitation and/or intrinsic nature of materials (Hou L. et al., 2015; Yuan et al., 2015). Superior electrochemical performance of the S-NFO indicates that the smaller subunits and high porosity play important roles in affecting the Li^+^ insertion/extraction process (see below for detailed discussions).

Figure 3D displays the typical cyclic voltammetry (CV) curves of S-NFO at a scan rate of 0.5 mV s^−1^, which is in accordance with the above charge-discharge profiles. A dominate reduction peak at 0.4 V is observed in the first cathodic sweep, which is assigned to the reduction of NiO and NiFe_2_O_4_ according to Equation (1, 2), and the formation of the SEI film (Guo et al., 2015; Chen et al., 2018). The weak and broad peak at 1.1 V might be due to the insertion of Li^+^ into electrode. In the following cycles, these two peaks shift to higher potential of 0.7 and 1.3 V, indicating irreversible capacity loss. Two peaks at 1.7 and 2.3 V in the anodic sweep are observed, which could be ascribed to the oxidation of Ni^0^ to Ni^2+^, and Fe^0^ to Fe^3+^, respectively Equations (3,4) (Guo et al., 2015; Chen et al., 2018). The highly overlapped CV curves in the subsequent cycles suggest a good reversibility of the electrochemical reactions.

(1) $$\begin{array}{r} NiO+2Li^{+}+2e^{-}\to Ni+Li_{2}O \end{array}$$

(2) $$\begin{array}{r} NiFe_{2}O_{4}+8Li^{+}+8e^{-}\to Ni+{2Fe+4Li}_{2}O \end{array}$$

(3) $$\begin{array}{r} Ni+Li_{2}O\to NiO+2Li^{+}+2e^{-} \end{array}$$

(4) $$\begin{array}{r} 2Fe+{3Li}_{2}O\to Fe_{2}O_{3}+6Li^{+}+6e^{-} \end{array}$$

Significantly improved rate performance is observed in the S-NFO in comparison with P-NFO and L-NFO benchmarks (Figure 3E). As the current density progressively increased from 1.0 to 10 A g^−1^, the S-NFO delivers high reversible capacities of 1292, 1130, 1004, 856, and 776 mAh g^−1^ at 1.0, 3.0, 5.0, 8.0, and 10 A g^−1^, respectively. Even at ultrahigh current densities of 15 and 20 A g^−1^, high capacities of 641 and 522 mAh g^−1^ are still observed. More importantly, as the current density drops to 1.0 A g^−1^, discharge capacity of the S-NFO gradually recovers to 1189 mAh g^−1^ as well. Apparently, the S-NFO shows high capacity retention of 92 % after 130 cycles at various current densities (from 1.0 to 20 A g^−1^). Such excellent rate performance outperforms not only P-NFO and L-NFO, but also compete with other NiFe_2_O_4_ or NiO electrodes (Park et al., 2015; Yu et al., 2015; Li C. et al., 2017) and some other TMOs/MTMOs hybrids (Zou et al., 2014; Hou L. et al., 2015; Yuan et al., 2015). Detailed rate capability comparison of this S-NFO with other NiFe_2_O_4_ and NiO based electrodes is illustrated in Figure 3F.

**Figure 4:**
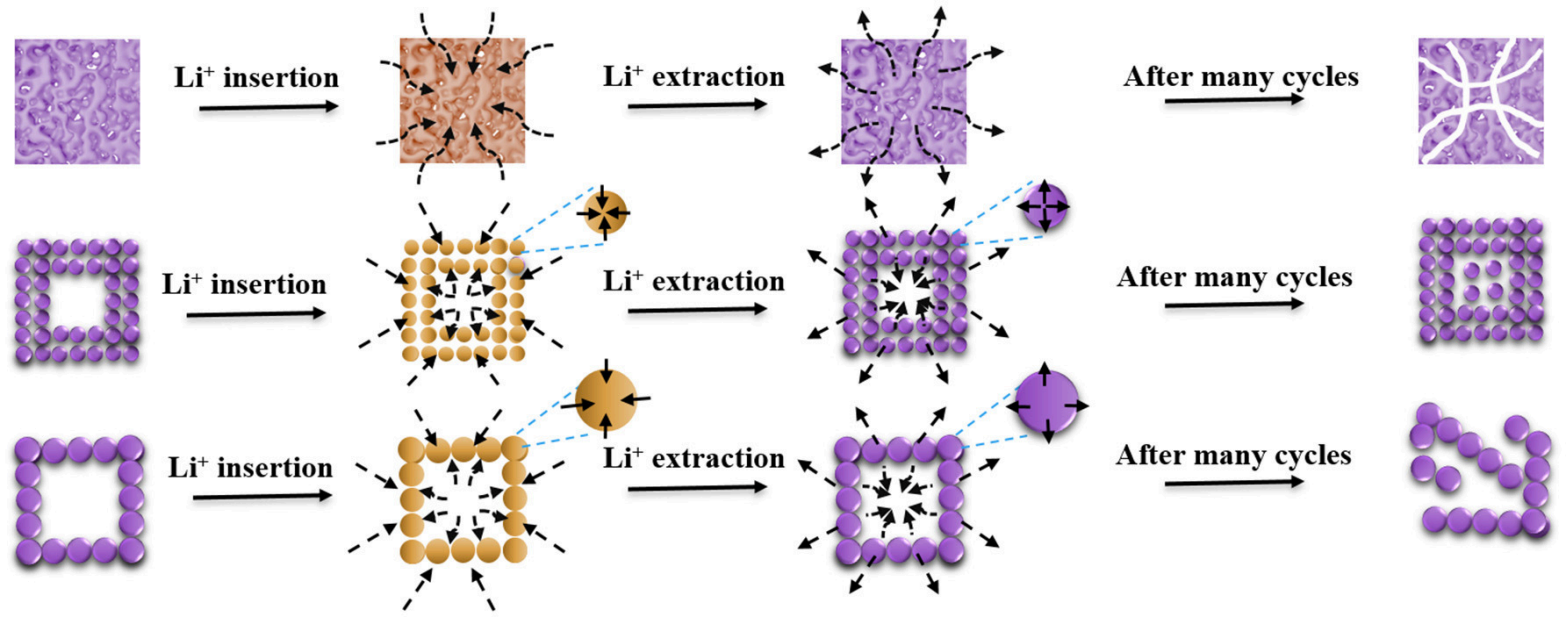
Illustration for the insertion and extraction channels of e^−^/Li^+^ in P-NFO, S-NFO, and L-NFO, and their corresponding structural evolution.

The distinct electrochemical performance of P-NFO, S-NFO, and L-NFO hetero-structures may be caused by their different porosity, particle size, and internal structure (Figure 4). For full utilization of active material, a better contact between electrolyte and electrode as well as a shorter path for Li^+^ transportation is needed. The hollow and hierarchical architecture of S-NFO simultaneously facilitates better electrolyte penetration and Li^+^ transportation. The small subunits of S-NFO not only effectively shorten the diffusion path of Li^+^ to improve the kinetics, but also provide more active sites for Li^+^ storage. Although the L-NFO also possesses a hierarchical hollow structure, its subunits are larger than the S-NFO, corresponding to a longer diffusion distance of Li^+^, and resulting in poor kinetics and low utilization of electrode, especially at high current densities. After Li^+^ insertion/extraction cycles, the small cavity in S-NFO may disappear due to the nanosize effect of conversion mechanism and the inevitable volume expansion. Fortunately, the initial small hollow in S-NFO can provide extra room for the inward volume expansion, resulting in a hierarchical cube with well dispersed subunits, which is helpful to stabilize the electrode structure. On the contrary, the lager hollow in L-NFO make the subunit near inner shell lack of confine from the interaction with each other, resulting in a continuous expanding during cycling and finally leading to a structure collapse (Cao et al., 2016). As for the P-NFO, similar volume expansion also occurs, but the filled internal in P-NFO cannot buffer such inward expansion, which causes also structural collapse after many repeated cycles.

**Figure 5:**
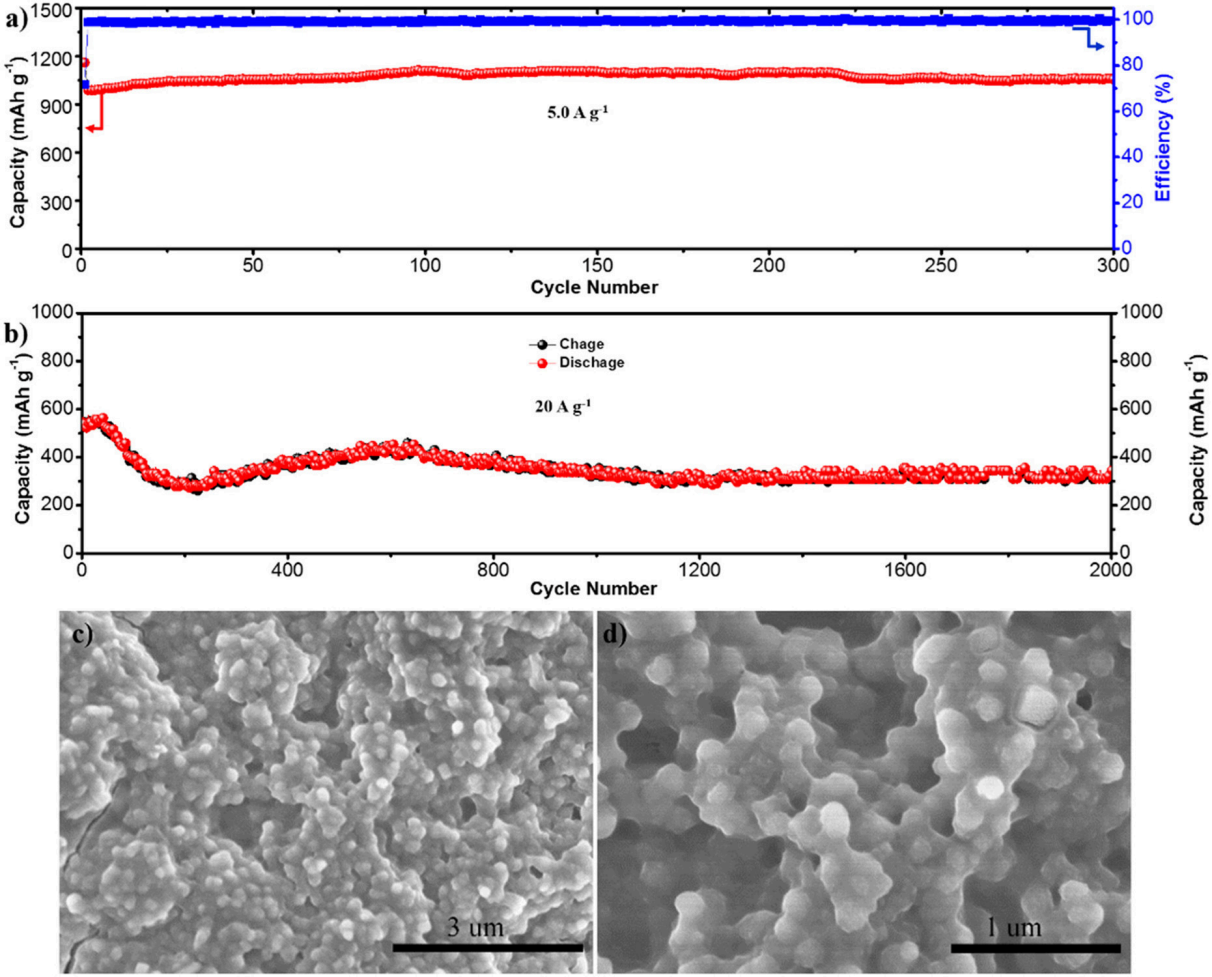
**(a,b)** Cycling stability of S-NFO electrode at 5.0 and 20 A g^−1^. **(c,d)** SEM images of the S-NFO electrode after 2,000 cycles.

Further characterizations about the cycle stability of S-NFO at high current densities are carried out (Figures 5a,b). At 5 A g^−1^, the S-NFO shows high capacity of 1,052 mAh g^−1^ after 300 cycles, with the CE stabilizes at almost 100 %, suggesting a good structural stability. More impressively, even at ultra-high current density of 20 A g^−1^, a high reversible capacity of 344 mAh g^−1^ is still delivered after 2,000 cycles, corresponding to only 0.018 % capacity decay per cycle. The rapid capacity decay during the initial 200 cycles and the continuous capacity growth in the following cycles are normally observed for conversion type anodes, which may be due to the reversible formation of a polymeric gel-like film and activation process (Jing et al., 2014). Structural stability of the S-NFO electrode is also investigated. As demonstrated in Figures 5c,d, the nanocube morphology is still well embedded in the electrode after 2,000 cycles and no obvious agglomeration or pulverization is observed after such long cycles. The corresponding post-cycled CV plots are presented in Figure S6, consistent with Figure 3D, implying the high electrochemical reversibility of S-NFO electrode. Therefore, it is reasonable to deduce that the small particle size and interior cavity of S-NFO can provide more room to buffer the volume changes during cycling, which benefits the structural stability and thus leads to better cycling performance.

**Figure 6:**
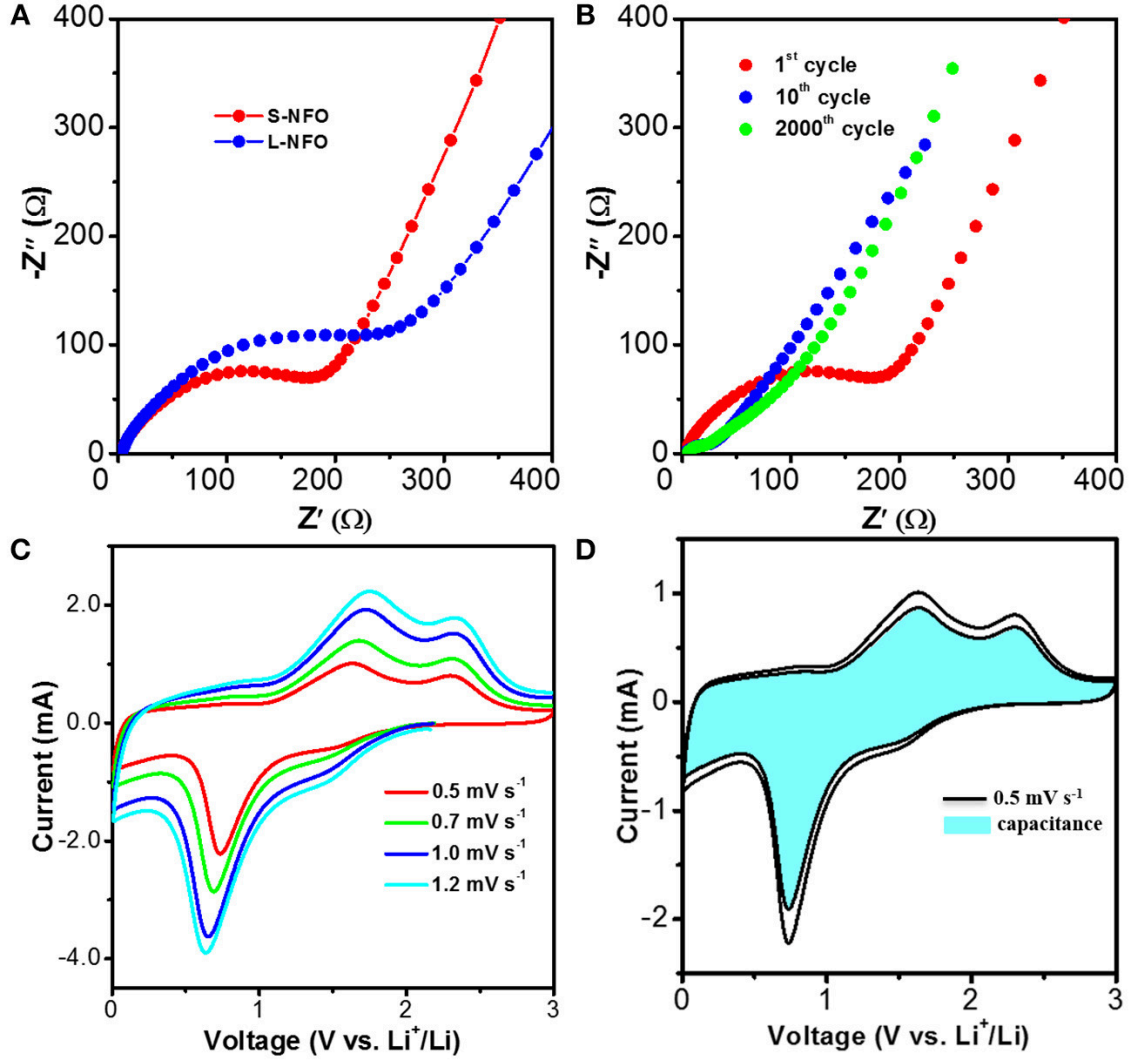
**(A)** Kinetics characterization of the as-assembled S-NFO, and L-NFO half-cells, at an open-potential of ~2.8 V, **(B)** Nyquist plots of S-NFO at the 1th, 10th, and 2000th cycle, tested after charging to 2.8 V. **(C)** CV curves of S-NFO at different sweep rate. **(D)** Capacitive contribution of S-NFO.

Effect of particle size on electrochemical kinetics of electrode is investigated by Nyquist plots (Figure 6A). The depressed semicircle in high frequency is related to charge transfer resistance of electrodes (R_ct_), and the inclined straight line in low frequency is associated with the mass transfer property. The R_ct_ value in S-NFO and L-NFO is calculated based on an equivalent circuit as shown in Figure S7, whereas R_s_, CPE, and W_o_ corresponds to the resistance of electrolyte, the constant phase elements, and the Warburg impedance, respectively. Clearly, the R_ct_ value of S-NFO electrode (192 Ω) is smaller than that of L-NFO (295 Ω), indicating faster kinetics of charge transfer. Therefore, small particle size can promote faster ion/electron transportation is confirmed, which is beneficial for good utilization of electrode at high currents. Figure 6B compares the Nyquist plot of the S-NFO after different cycles. The decreased R_ct_ in the initial ten cycles may be ascribed to the formation of a conductive intermediate (Hu et al., 2015). No obvious change is found between the 10th and 2000th cycles (Figure S8), indicating the well preservation of kinetic superiority after the long-term cycles. The larger R_ct_ of L-NFO is in consistent with its structure, which features longer diffusion distance, as illustrated in Figure 4. Moreover, there is a profound difference among the phase angel of S-NFO, and L-NFO. Generally, the more vertical the phase angle is, the more capacitive behavior there is. Therefore, it is reasonable to speculate the existence of capacitive behavior in the S-NFO electrode.

The CV curves of S-NFO electrode at different sweep rates (0.5 ~ 1.2 mV s^−1^, Figure 6C) are used to determine its capacitive behavior. As shown in Figure 6D, about 86% faction of the total charge roots from the capacitive process according to the calculation method previously reported (Wang et al., 2016). Such high percentage of capacitance behavior contributes to the excellent rate performance and cycle stability of S-NFO. The extraordinary electrochemical performance of this S-NFO demonstrates its huge potential for fast Li ion storage.

**Figure 7:**
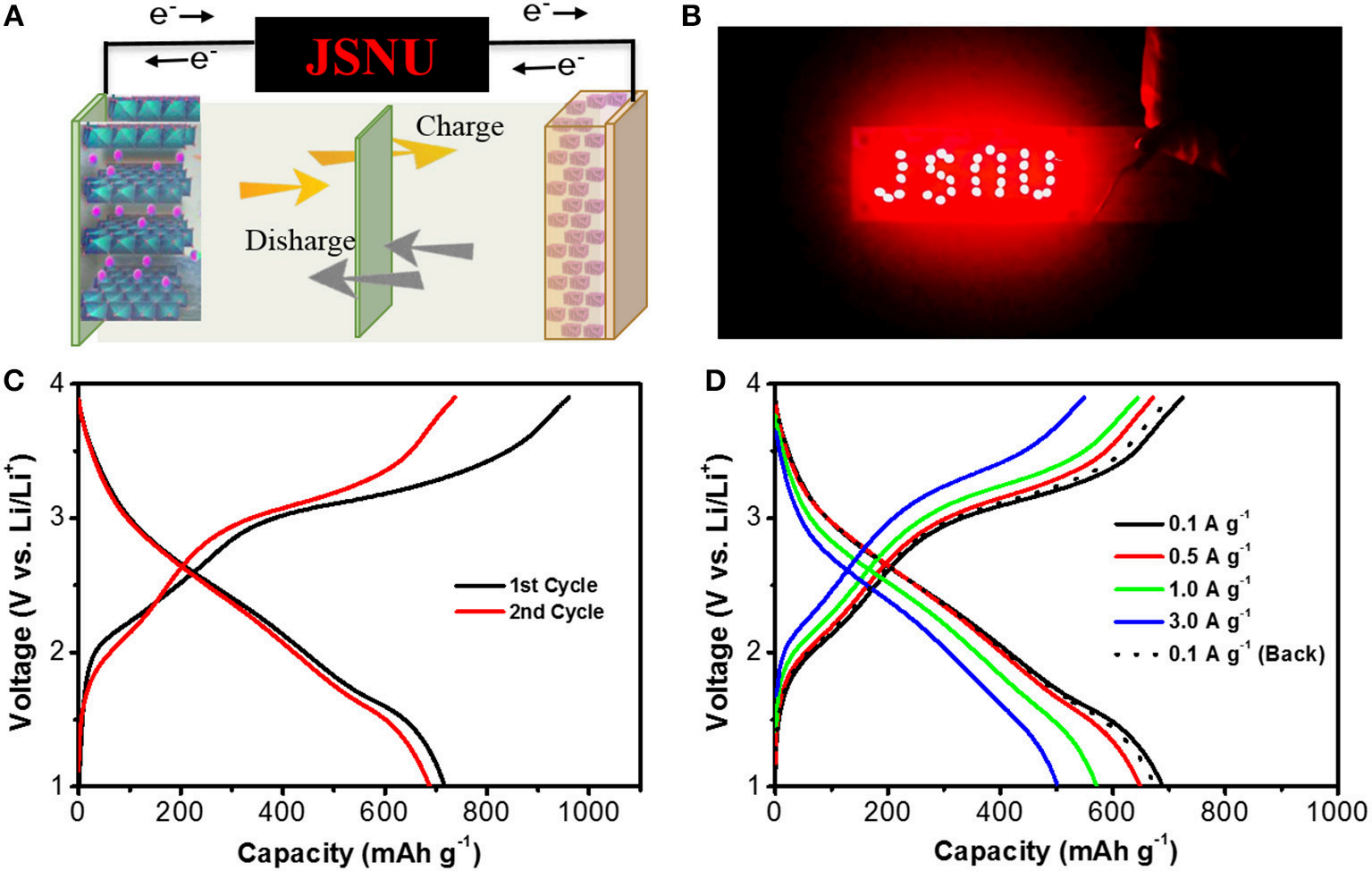
**(A)** Illustration of the full cell using LiCoO_2_ as cathode and S-NFO as anode. **(B)** Digital photo of an LED array that is powered by the S-NFO based full cell. **(C,D)** Charge-discharge plots of the S-NFO based full-cells at 0.1 A g^−1^ and at various current densities of 0.1, 0.5, 1.0, 3.0 A g^−1^, respectively.

To verify the practical application of this S-NFO, full-cells are assembled by using the S-NFO as anode and commercial LiCoO_2_ as cathode, as demonstrated in Figure 7A. An LED array with JSNU logo that is consisted of 33 red LEDs in parallel was powered by an S-NFO based full-cell. As shown in Figure 7B, the whole LED array could be easily lighted up, demonstrating the viability and practical applications of this S-NFO electrode material. Charge-discharge curves of the full-cells at 0.1 A g^−1^ in a voltage range of 1.0–3.9 V were presented in Figure 7C. In the first cycle, a charge/discharge capacity of 961/737 mAh g^−1^ is delivered, corresponding to an ICE value of 76.7%. About 97.2% capacity was retained in the second cycle. Rate performance of the full-cells was tested at various current densities, as shown in Figure 7D. When increasing the current density from 0.1 to 3.0 A g^−1^, a high reversible capacity of 649, 572, and 500 mAh g^−1^ was delivered at 0.5, 1.0 and 3.0 A g^−1^, respectively. As the current density drops back to 0.1 A g^−1^, 98.1% capacity was recovered, implying the high reversibility of full-cells. Cycling performance of the full cells was carried out at 0.5 A g^−1^ after activating at 0.1 A g^−1^ for three cycles (Figure S9), which shows a reversible capacity of 300 mAh g^−1^ at the end of 50 cycles.

## Conclusion

In summary, by combining the advantages of hetero-structure and nano-architecture, the electrochemical performance of NiFe_2_O_4_/NiO hybrid material is significantly improved. A series of NiO/NiFe_2_O_4_ hetero-structures with different porosity, particle size, and shell/internal structures has been synthesized and investigated as anode materials for ultra-fast LIBs. Experimental results highlights the interactions between structural geometry and electrochemical behavior, demonstrating that hetero-structure phase, small particles size, and high porosity offer obvious advantages in improving the Li^+^ storage property. By optimizing these factors, faster electrochemical kinetics and long-term cycling stability have been achieved in the S-NFO electrode. Impressive rate performance and cycle stability at high current densities are observed in the S-NFO sample, which retains 1,052 and 344 mAh g^−1^ after 300 and 2,000 cycles at 5.0 and 20 A g^−1^, respectively. The post-cycled SEM images demonstrate the well-reserved structural durability of this unique electrode. The results here may shed light on further design and fabrication of other high performance micro-nanostructured materials for energy storage and conversion technologies.

## Author Contributions

YingW conducted the experiments and helped writing the manuscript. SW helped operating experiments and data analysis. YijingW, CW, and XH supervised this research work. All authors read and approved the final manuscript.

### Conflict of Interest Statement

The authors declare that the research was conducted in the absence of any commercial or financial relationships that could be construed as a potential conflict of interest.
