# Supplementary material for: Morphology Controllable Synthesis of NiO/NiFe2O4 Hetero-Structures for Ultrafast Lithium-Ion Battery
Source: Front Chem. 2019 Jan 10;6:654. doi: 10.3389/fchem.2018.00654 (PMC6335950; doi:10.3389/fchem.2018.00654)
Supplement: Supplementary file 1 [file Table_1.docx]

Supplementary Material

Morphology controllable synthesis of NiO/NiFe_2_O_4_ hetero-structures for ultrafast lithium-ion battery

***Ying Wang,^1^ Shengxiang Wu,^1^ Chao Wang,^1^* Yijing Wang^2^* Xiaopeng Han^2,3^****

^1^School of Chemistry & Materials Science, Jiangsu Key Laboratory of Green Synthetic Chemistry for Functional Materials, Jiangsu Normal University, Xuzhou, Jiangsu 221116, China.

^2^Key Laboratory of Advanced Energy Materials Chemistry (MOE), College of Chemistry, Nankai University, Tianjin 300071, China.

^3^School of Materials Science and Engineering, Tianjin Key Laboratory of Composite and Functional Materials, Tianjin University, Tianjin 300072, China.

*** Correspondence:**

Chao Wang
wangc@jsnu.edu.cn

Yijing Wang
wangyj@nankai.edu.cn

Xiaopeng Han
xphan@tju.edu.cn


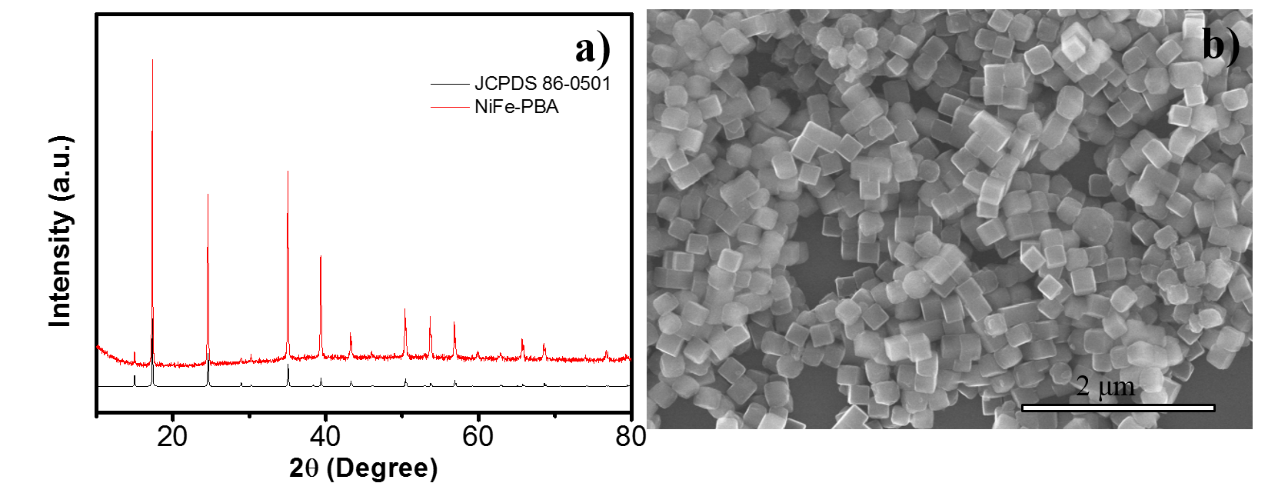


**Figure S1** (a) XRD pattern and (b) SEM images of the NiFe-PBA


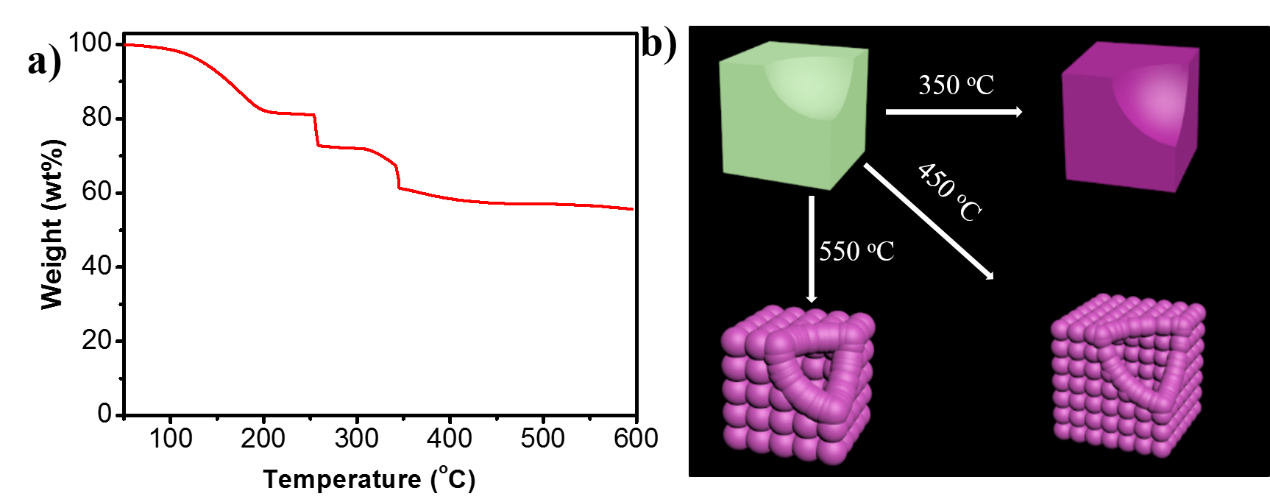


**Figure S2** (a) TGA curve of the NiFe-PBA, ramp: 10 °C min^-1^ in air, and (b) illustration for the synthesis of morphology controllable NiO/NiFe_2_O_4_hetero-structures.

**
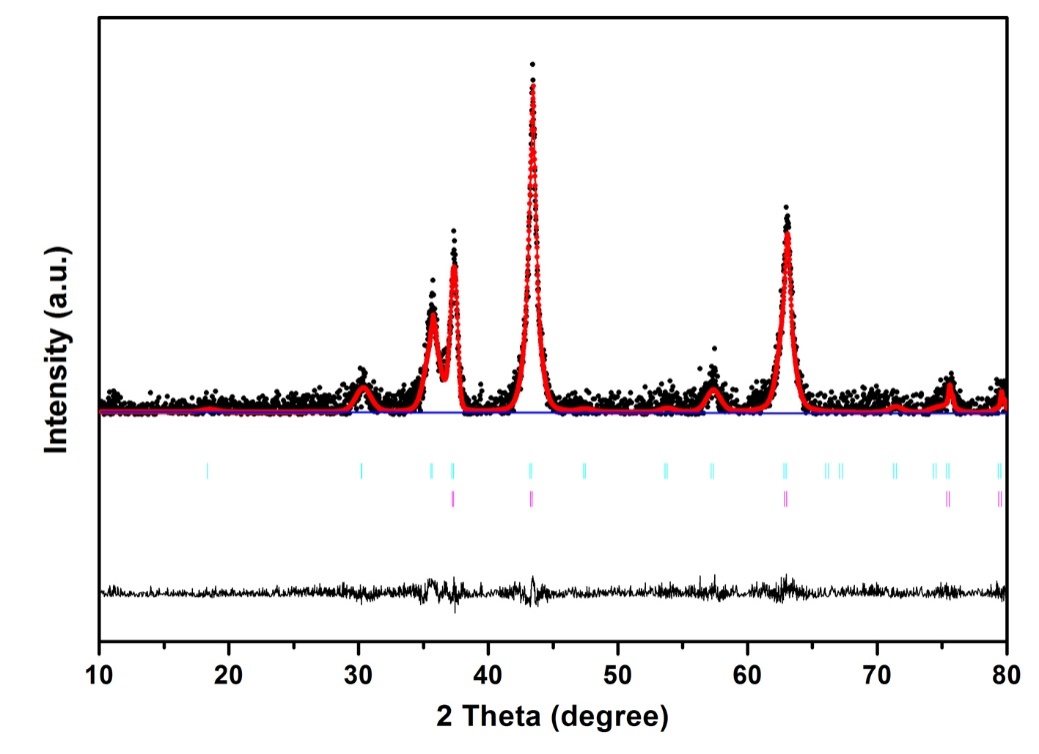
**

**Figure S3**. Rietveld refinement of the XRD pattern of the S-NFO,with experimental data (black dots), calculated curves (red line), position of allowed Bragg reflections (cyan vertical bars for NiFe_2_O_4_ and pink vertical bars for NiO), and difference profile (black line). The Rietveld refinement is calculated by using a GSAS software. The phase content of NiFe_2_O_4_ and NiO is calculated to be 63 % and 37%.


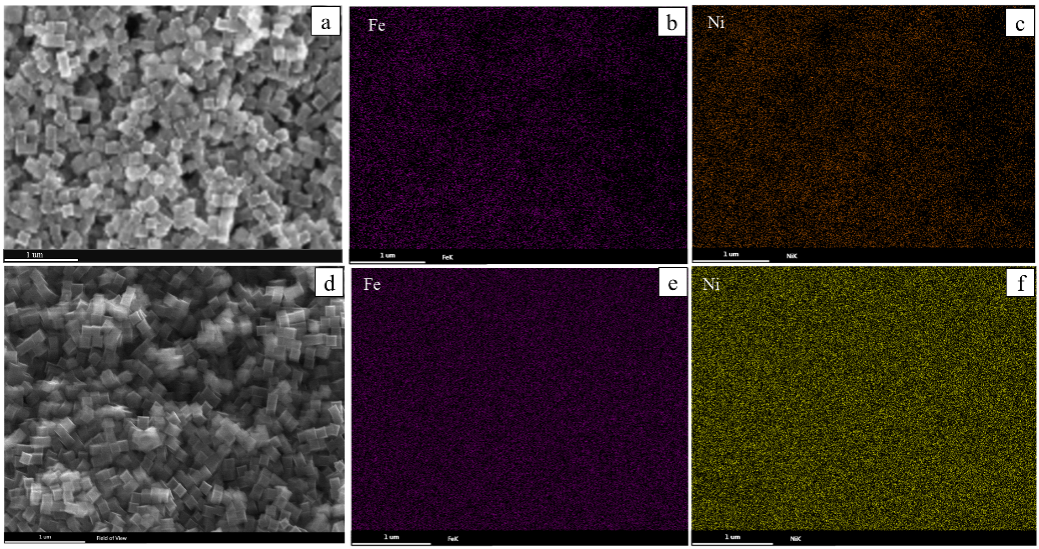


**Figure S4** SEM-EDS elemental distribution of (a-c) the P-NFO, and (d-f) the L-NFO.


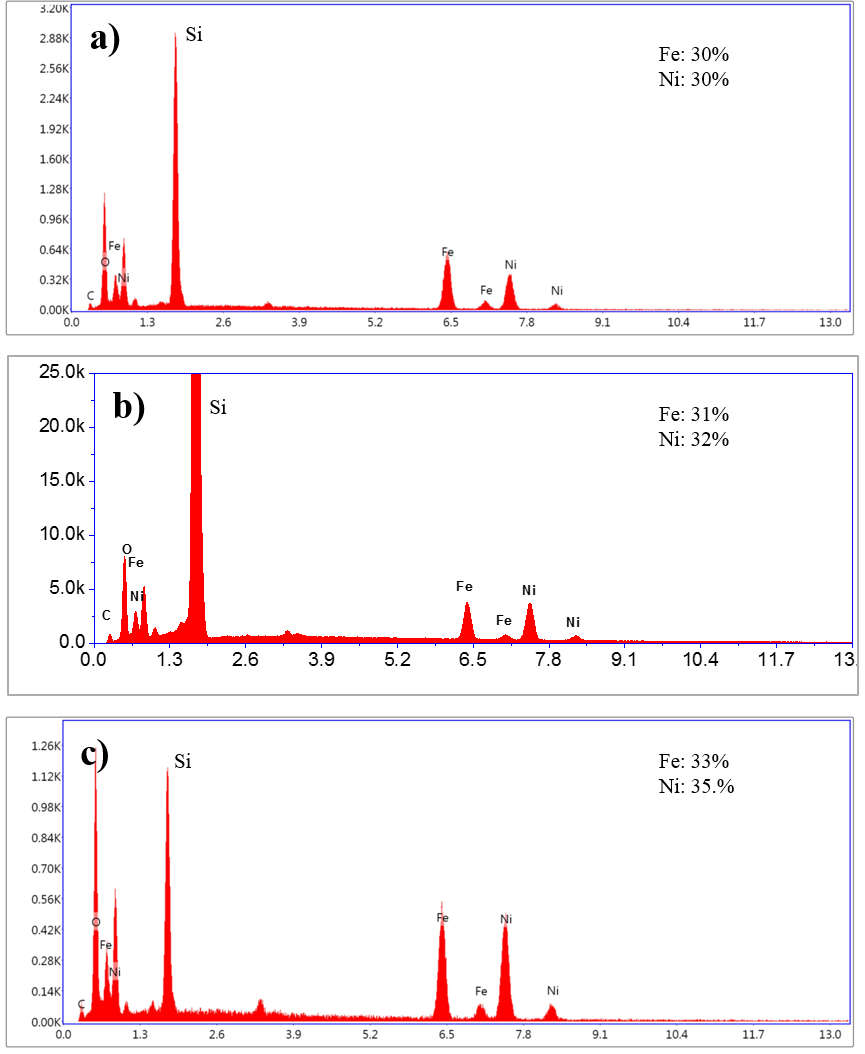


**Figure S5** SEM-EDS plots for (a) P-NFO, (b) S-NFO, and (c) L-NFO. Si peaks are collected from the substrate, and the weight ratio of Ni and Fe are calculated based on only Ni, Fe, and O.


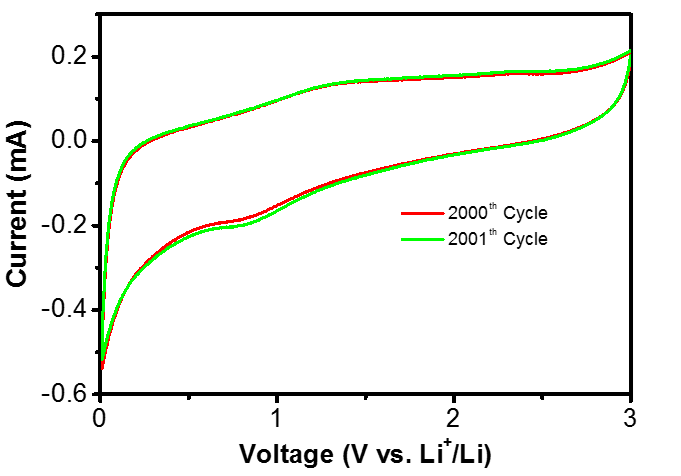


**Figure S6** CV curves of the S-NFO electrode at the 2000^th^ and 2001^th^ cycle with a sweep rate of 0.5 mV s^-1^.


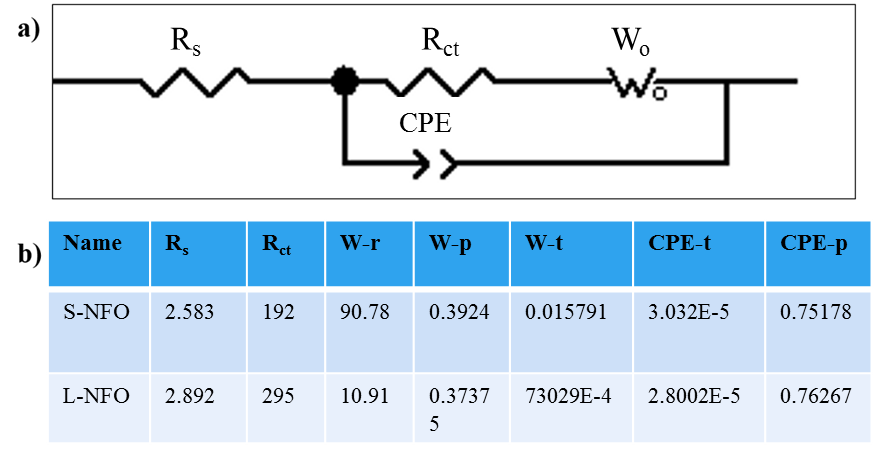


**Figure S7** (a) Equivalent circuit for the Nyquist plots, (b) list of the fitting results.


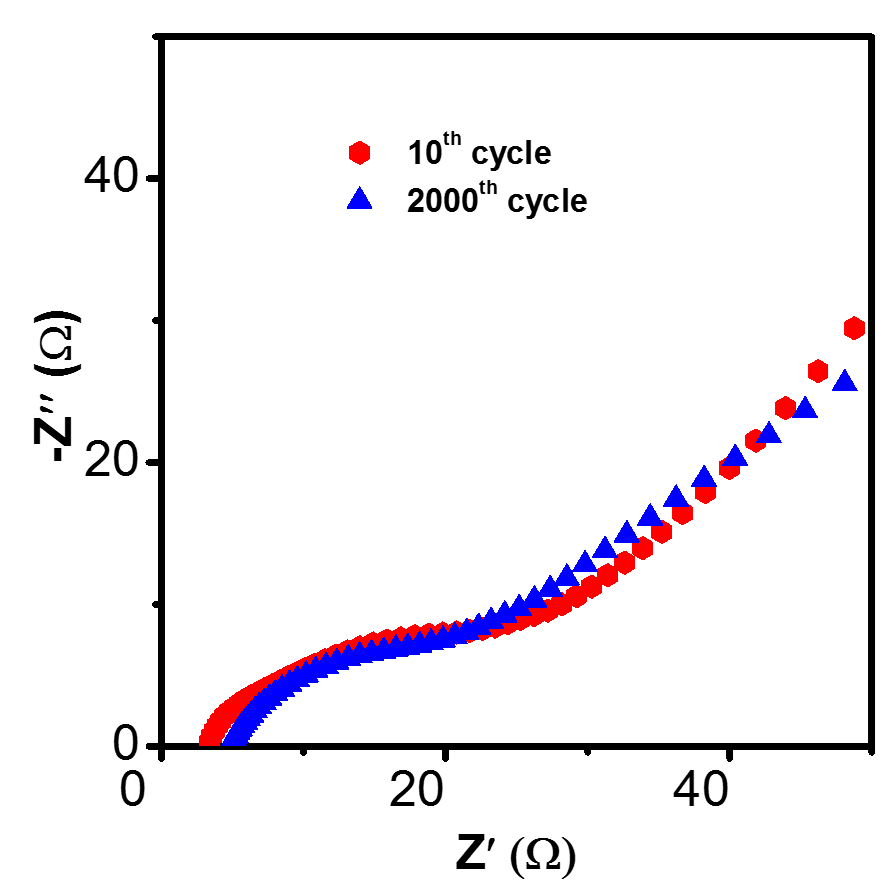


**Figure S8** Enlarged Nyquist plots of S-NFO electrode at the 10^th^ and 2000^th^ cycle.

**Figure S9** Cycling performance of the S-NFO based full-cells at a current density of 0.5 A g^-1^, tested after activating at 0.1 A g^-1^ for three cycles.
